# Case report of a novel mutation in the *TNC* gene in Chinese patients with nonsyndromic hearing loss

**DOI:** 10.1097/MD.0000000000037702

**Published:** 2024-04-19

**Authors:** Shouxia Li, Shurui Li, Dingli Chen, Subin Zhao, Cairu Liu, Ruimin Zhang, Yongxue Chen, Xiangrui Guo, Xuedong Song

**Affiliations:** a Department of Laboratory Medicine, Handan Central Hospital, Hebei Medical University, Handan, Hebei, China; b Department of General Surgery, Affiliated Hospital of Hebei University of Engineering, Handan, Hebei, China; c Department of Obstetrics, Handan Central Hospital, Hebei Medical University, Handan, Hebei, China; d Department of Neonatology, Handan Central Hospital, Hebei Medical University, Handan, Hebei, China; e Department of Anesthesiology, Handan Central Hospital, Hebei Medical University, Handan, Hebei, China; f Department of Laboratory Medicine, Shandong Provincial Hospital Affiliated to Shandong First Medical University, Jinan, Shandong, China.

**Keywords:** mutation analysis, nonsyndromic hearing loss, protein structure prediction, *TBC1D24* gene, *TNC* gene

## Abstract

**Rationale:**

Hereditary hearing loss is known to exhibit a significant degree of genetic heterogeneity. Herein, we present a case report of a novel mutation in the tenascin-C (*TNC*) gene in Chinese patients with nonsyndromic hearing loss (NSHL).

**Patient concerns:**

This includes a young deaf couple and their 2-year-old baby.

**Diagnoses:**

Based on the clinical information, hearing test, metagenomic next-generation sequencing (mNGS), Sanger sequencing, protein function and structure analysis, and model prediction, in our case, the study results revealed 2 heterozygous mutations in the *TNC* gene (*c.2852C>T, p.Thr951Ile*) and the TBC1 domain family member 24 (*TBC1D24*) gene (*c.1570C>T, p.Arg524Trp*). These mutations may be responsible for the hearing loss observed in this family. Notably, the heterozygous mutations in the *TNC* gene (*c.2852C>T, p.Thr951Ile*) have not been previously reported in the literature.

**Interventions:**

Avoid taking drugs that can cause deafness, wearing hearing AIDS, and cochlear implants.

**Outcomes:**

Regular follow-up of family members is ongoing.

**Lessons:**

The genetic diagnosis of NSHL holds significant importance as it helps in making informed treatment decisions, providing prognostic information, and offering genetic counseling for the patient’s family.

## 1. Introduction

A person who is not able to hear as well as someone with normal hearing is said to have hearing loss. Loss of hearing, if not identified and addressed, can have far-reaching consequences, adversely affecting language development, psychosocial well-being, quality of life, educational attainment, and economic independence at various stages of life.^[1–3]^ The Global Burden of Disease Study measured years lived with disability and found that hearing loss is the fourth leading cause of disability globally.^[4]^ The World Health Organization (WHO) estimates that over 400 million, including 34 million children, live with disabling hearing loss, affecting their health and quality of life (https://www.who.int/en/news-room/fact-sheets/detail/deafness-and-hearing-loss, accessed September 3, 2023). Meanwhile, WHO predicts that by 2050 over 700 million people will have disabling hearing loss.

Approximately 50% of hearing loss is attributed to genetic factors, with 70% of hereditary hearing loss falling under the category of nonsyndromic hearing loss (NSHL).^[5]^ NSHL refers to hearing loss that is not associated with any other underlying diseases. The genetic diagnosis of NSHL holds significant importance as it helps in making informed treatment decisions, providing prognostic information, and offering genetic counseling for the patient’s family.^[6,7]^ According to the Morl Lab at the University of Iowa, to date, 224 genes have been reported to be associated with hearing loss (https://morl.lab.uiowa.edu/genes-included-otoscope-v9). Additionally, the Hereditary Hearing Loss Homepage reports that there were 124 genes linked to NSHL (https://hereditaryhearingloss.org/), accessed on October 9, 2023. NSHL is known to have 4 genetic patterns: autosomal dominant, autosomal recessive, X-linked, and mitochondrial.^[8]^ However, a significant portion of sensorineural hearing loss still lacks a known genetic explanation.^[9]^

For many decades, linkage analysis combined with Sanger sequencing has been the most powerful and widely used strategy to identify the genes responsible for Mendelian diseases.^[10]^ In recent times, advancements in molecular diagnostic technology have significantly reduced testing costs, leading to the emergence of metagenomic next-generation sequencing (mNGS) as an effective and comprehensive method for diagnosing NSHL.^[11–14]^ In this study, we employed mNGS, Sanger sequencing, protein function, and structure analysis, and model prediction to investigate and analyze the molecular biology of a Chinese family affected by nonsyndromic deafness.

## 2. Case report

**Figure 1.**
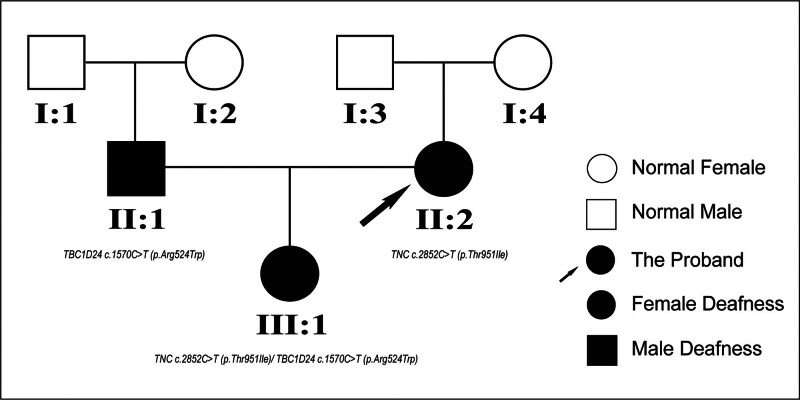
The genealogical tree of the Chinese pedigree. The black arrow indicates the proband (II:2) of the family, and the filled symbols represent affected members.

The proband (II-2) and her family members comprised 7 members in 3 generations (Fig. 1). The family was ascertained from the Hebei Province of China. The parents of the proband had normal hearing screening, no family history, nonconsanguineous marriage, and no history of infection or toxic drug exposure during pregnancy. All the procedures of investigation were approved by the Ethics Reviewing Committee of Handan Central Hospital and were carried out only after obtaining written informed consent from each participant or family.

The proband (II-2) was a 28-year-old pregnant woman with NSHL and who was admitted to the hospital waiting for the baby. After asking her husband was also a deaf patient, the young deaf couple welcomed their first baby (III-1) on August 29, 2021. However, obstetricians performed an otoacoustic emission test within 48 hours and found that no waveform hearing was elicited in both ears of the newborn, and the hearing screening failed. According to the genetic counseling needs of the family, mNGS of inherited deafness gene exons in peripheral blood of the proband (II-2), her husband (II-1), and newborn (III-1) were performed on September 2, 2021. On postpartum day 42, we did a return visit and still found that no waveform hearing was elicited in both ears of the newborn (III-1). The girl (III-1) was 2 years old and was living with grandma and grandpa, we conducted the fourth return visit in August 2023. The girl (III-1) showed moderate deafness in the low-frequency area, normal vision, and no other system abnormalities The results of brainstem evoked potential showed that the waveforms of both sides were poorly differentiated and the repeatability was poor.

**Figure 2.**
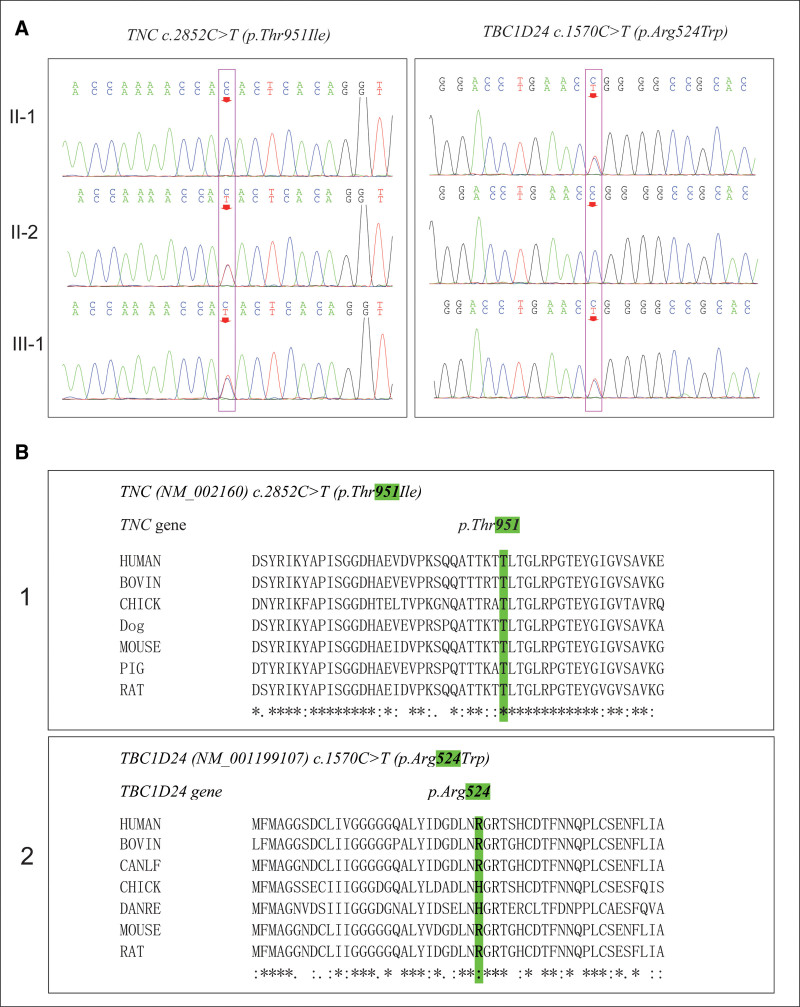
Sanger sequencing validation and conservation analysis among different species. (A)*TNC c.2852C>T (p.Thr951Ile*) and *TBC1D24 c.1570C>T (p.Arg524Trp*) mutations in the proband and her family members were verified by Sanger sequencing. Red arrows indicate the position of the nucleotide changes identified in this study. II-2, III-1 carried the *c.2852C>T (p.Thr951Ile*) mutation in *TNC*, and II-1, III-1 carried the *c.1570C>T (p.Arg524Trp*) mutation in *TBC1D24.* (B). Multiple sequence alignment showed that *p.Thr951* and *p.Arg524* are positioned in a highly conserved region. Changes in amino acids are highlighted in the green boxes. “*”: a completely consistent residue, “.” residues with weak and similar properties, “:” residues with very similar properties.

The NGS analysis and Sanger sequencing validation showed heterozygous missense mutation *c.2852C>T (p.Thr951Ile*), located in the eighth exon domain of the tenascin-C (*TNC*) gene (*NM_002160*) in the proband (II:2), heterozygous missense mutation *c.1570C>T (p.Arg524Trp*), located in the eighth exon domain of the TBC1 domain family member 24 (*TBC1D24*) gene (*NM_001199107*) in the husband (II:1) of the proband. Meanwhile, the newborn (III-1) was found to have a heterozygous mutation of *TNC* with *c.2852C>T* from the proband (II-2) and a *TBC1D24* with *c.1570C>T* from the father (II-1) (Fig. 2A). No mutations were found in other deafness genes, and no clinically significant mitochondrial gene variation and copy number variation were found. Gene conservation analysis is a method used to study genetic differences and evolutionary relationships among different species (Fig. 2B). The amino acids (*p.Thr951*) in *TNC* protein are conserved across in the conservation analysis of species alignment.

Different bioinformatics tools were used for the prediction of protein function damage, as shown in Table 1. The heterozygous missense variant of *c.2852C>T*, results in an amino acid sequence changed from *Thr* to *Ile* at position 951 of the *TNC*. Sorting intolerant from tolerant (SIFT) gave a score of 0.03, suggesting that the site was considered a “Deleterious” variation. PolyPhen2 gave a score of 0.994, suggesting that the site might be a damaging variation. MutationTaster showed “disease_causing,” and a prediction accuracy of 0.9998 for this missense mutation. Meanwhile, the heterozygous missense variant of *c.1570C>T*, which results in an amino acid sequence changed from *Arg* to *Trp* at position 524 of the *TBC1D24*. SIFT gave a score of 0.22, suggesting that the site was considered a “Tolerated” variation. PolyPhen2 gave a score of 0.011, suggesting that the site might be a benign variation. MutationTaster showed “disease_causing,” and a prediction accuracy of 0.9972 for this missense mutation. The *TNC* variant *c.2852C>T* present in the proband had not previously been reported in individuals. We extrapolated this to be a potential pathogenic variant and a novel variant associated with hearing loss in a Chinese individual.

**Table 1:** Variants analysis of the NSHL patients in this study.

Gene (RefSeq, chromosome)	Nucleotide changes	Amino acid changes	Exon	Type of variation	Classification of variation	GnomAD-exome	ExAC	ACMG rating	Prediction of protein function damage	Pathogenicity reports
*TNC* (NM_002160, chr9:117838677)	c.2852*C>T*	p.Thr951Ile	Exon 8	Heterozygous	Missense variants	0.0	0.0	Unclear clinical significance (PM1, PP3)	SIFT (Deleterious), Polyphen2 (Possibly damaging), MutationTaster (disease_causing)	The variation has not been reported in the literature
TBC1D24 (NM_001199107, chr16:2550849)	c.1570*C>T*	p.Arg524Trp	Exon 8	Heterozygous	Missense variants	0.0	0.0	Unclear clinical significance (PM1, PM2, PP3)	SIFT (Tolerated), Polyphen2 (Benign), MutationTaster (disease_causing)	National Center for Biotechnology Information. ClinVar; [VCV000227980.38], https://www.ncbi.nlm.nih.gov/clinvar/variation/VCV000227980.38 (accessed October 8, 2023)

Reference databases of pathogenicity reports: HGMD Pro, PubMed, and ClinVar. Reference databases for prediction of protein function damage: SIFT, Polyphen2, and MutationT.

ACMG = The American College of Medical Genetics and Genomics, NSHL = nonsyndromic hearing loss, TBC1D24 = *TBC1* domain family member 24, TNC = tenascin-C.

**Figure 3.**
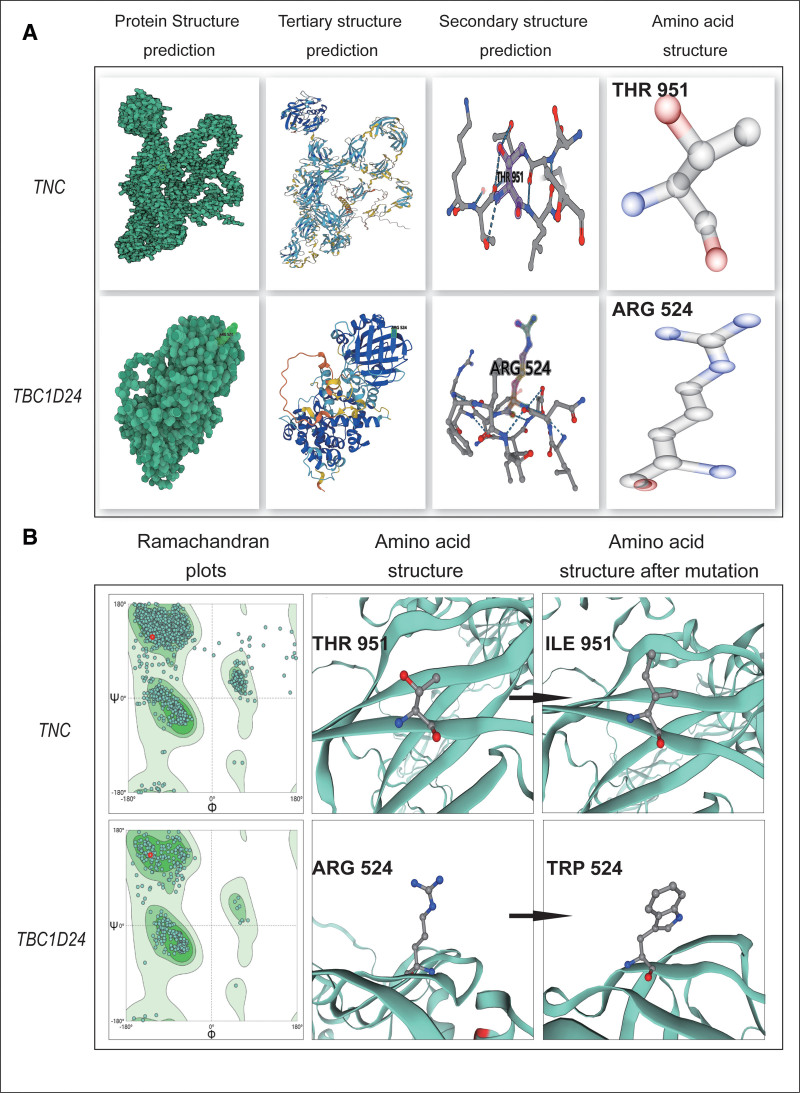
*TNC c.2852C>T (p.Thr951Ile*) and *TBC1D24 c.1570C>T (p.Arg524Trp*) mutations 3D protein modeling analysis. (A) The 3D overall picture of *TNC* protein and *TBC1D24* protein were modeled using AlphaFold online prediction program. From left to right, the normal protein structure prediction model, Tertiary structure prediction model, Secondary structure prediction model and amino acid structure model are constructed. (B) The 3D overall picture of mutation *TNC* protein and *TBC1D24* protein were modeled using the SWISS model. From left to right, Ramachandran plots, amino acid structure and amino acid structure after mutation are constructed.

To better understand the structural and functional impact of heterozygous missense variant on protein and the potential contribution to the etiology of hearing loss, the human *TNC* protein (951 amino acids) and *TBC1D24* protein (524 amino acids) were modeled using AlphaFold online prediction program (https://alphafold.ebi.ac.uk/, Fig. 3A) and SWISS Model (http://swissmodel.expasy.org/, Fig. 3B), The model covered the target sequence of *TNC* and *TBC1D24* protein. As shown in Figure 3B, we can visualize that the amino acid sequence changed from Thr to Ile at position 951 of the TNC protein 3D structure, and from *Arg* to *Trp* at position 524 of the *TBC1D24* protein 3D structure. The amino acids (*p.Thr951*) in *TNC* protein are conserved across in the conservation analysis of species alignment (Fig. 2B). Different amino acids have different structures, and the chemical bonds around amino acids may also change after the mutation of amino acids, which affects the change of protein structure and then affect the protein function. Therefore, the changes in amino acid sequences may seriously impact the normal development of hearing.

## 3. Discussions

Hereditary hearing loss is known to exhibit a significant degree of genetic heterogeneity.^[5]^ This is evident in the wide range of deafness phenotypes, inheritance patterns, and pathogenic genes associated with the condition. In our case, the study results revealed 2 heterozygous mutations in the *TNC* gene (*c.2852C>T, p.Thr951Ile*) and the *TBC1D24* gene (*c.1570C>T, p.Arg524Trp*) (Table 1). These mutations may be responsible for the hearing loss observed in this family. Notably, the heterozygous mutations in the *TNC* gene (*c.2852C>T, p.Thr951Ile*) have not been previously reported in the literature.

*TNC*, a gene placed on chromosome 9q33.1, produces an 8.5-kb transcript translated into an extracellular matrix glycoprotein *TNC*,^[15]^ and the *TNC* protein is a multifunctional hexameric glycoprotein that plays a regulatory role during development, tissue remodeling, and disease.^[16]^ In the case of hearing loss, *TNC* has recently been found to be causative for DFNA56 (OMIM: 615629), and *TNC* expresses under the basilar membrane in the cochlea, and is important for auditory development and self-recovery from injuries.^[17]^

To date, only 3 *TNC* variants have been described as causing DFNA56. Yali Zhao et al have reported 2 mutations *TNC c.5368A > T (p.Thr1796Ser*) and *TNC c.5317G > A (p.Val1773Met*) in exon 19 of *TNC* in Chinese deafness families.^[17]^ In 2022, researchers Jin^[18]^ reported the *TNC* mutation *c.1641C > A (p.Cys547X*) in exon 3 of TNC in Chinese deafness families. In our case, a novel heterozygous mutation *c.2852C>T (p.Thr951Ile*) of the *TNC* gene in Chinese deafness families was not reported in the literature, which enriched the known *TNC* variant spectrum in HGMD Pro and PubMed databases. The heterozygous missense variant of *c.2852C>T*, results in an amino acid sequence changed from *Thr* to *Ile* at position 951 of the *TNC*. Despite the American College of Medical Genetics and Genomics Rating showing unclear clinical significance (PM1, PP3) for mutation *c.2852C>T (p.Thr951Ile*) of the *TNC* gene. However, SIFT gave a score of 0.03, suggesting that the site was considered a deleterious variation. PolyPhen2 gave a score of 0.994, suggesting that the site may be a damaging variation. MutationTaster showed a disease-causing and predictive accuracy of 0.9998 for this missense mutation. Therefore, *TNC c.2852C>T (p.Thr951Ile*) may give rise to hearing loss present in the proband and her baby. The underlying molecular pathomechanism of DFNA56 is still unknown and needs to do more in-depth research.

Located at 16p13.3, *TBC1D24* encodes a member of the TBC family domain proteins. *TBC1D24* protein is a binding partner of ARF6 (ADP ribosylation factor 6), which regulates dendritic branching, spine formation, and axonal extension.^[19]^ The first ADHL-related *TBC1D24* pathogenic variant (*p.Ser178Leu*) was also identified in 2014, found in parallel in a European and a Chinese family.^[20,21]^ Prior to 2020, no other *TBC1D24* alteration has been associated with this condition. The second *TBC1D24*-related ADHL variant (*p.Asn307His*) has been reported recently in a study of 2 unrelated HL families from Austria and the UK.^[22]^

In our case, the other heterozygous missense mutation *c.1570C>T (p.Arg524Trp*), which results in an amino acid sequence changed from *Arg* to *Trp* at position 524, located in the eighth exon domain of the *TBC1D24* gene in the proband husband. SIFT gave a score of 0.22, suggesting that the site was considered a tolerated variation. PolyPhen2 gave a score of 0.011, suggesting that the site might be a benign variation. MutationTaster showed disease-causing, and prediction accuracy of 0.9972 for this missense mutation. The American College of Medical Genetics and Genomics rating showed unclear clinical significance (PM1, PM2, PP3). To date, *TBC1D24 c.1570C>T (p.Arg524Trp*) of the gene has been reported 3 times uncertain significance and 4 times likely benign in literature, conflicting interpretations of pathogenicity (https://www.ncbi.nlm.nih.gov/clinvar/variation/VCV000227980.38 (accessed October 8, 2023). This is also confirmed in our report.

Different amino acids have different structures, and the chemical bonds around amino acids may also change after the mutation of amino acids, which affects the change of protein structure and then affects protein function.^[23–26]^ The 3D model was constructed for structural analysis of WT/Mut *TNC* and *TBC1D24* proteins to determine the pathogenicity of mutant *TNC* and *TBC1D24* according to SWISS MODEL and AlphaFold online prediction program. Structural modeling demonstrated that the *p.Thr951Ile* and *p.Arg524Trp* variants altered the normal protein structure (Fig. 3). When amino acid 951 is changed to isoleucine, *TNC* side chains also change and tend to be longer than those of a structure in which amino acid 951 is threonine (Fig. 3B). The *TBC1D24* variant has a different side chain than the wild-type protein due to the substitution of heterocyclic tryptophan for aliphatic arginine (Fig. 3B). In addition, bioinformatics analysis using Mutation Taster and SIFT supported the pathogenicity hypothesis of both variants. Therefore, heterozygous mutations were predicted to affect the amino acid side chain. The disruption of *TNC* and *TBC1D24* protein function and interactions with other molecules and residues could have far-reaching consequences for normal hearing development in this family, potentially leading to serious issues.

Hearing is a key component of human intrinsic capacity; it is the sense most relied upon to communicate and engage with others. Any decline in hearing capacity at any point during the life course, if not addressed promptly, can adversely affect day-to-day functioning.^[27,28]^ Nowadays, the development of molecular diagnostic technology has greatly reduced the cost of testing, and metagenomic next-generation sequencing has become an effective way of providing comprehensive and efficient diagnosis for hearing loss.^[11–14]^ To find out the mechanism of hearing loss, timely intervention measures should be taken, such as avoiding taking drugs that can cause deafness, wearing hearing AIDS, and cochlear implants, so that they can leave the silent world as soon as possible and return to normal life.

## 4. Conclusion

We have identified a novel variant *c.1570C>T (p.Arg524Trp*) in the *TNC* gene in a Chinese family, further expanding our knowledge of the *TNC* variant spectrum.

## Acknowledgments

We thank the proband and her family for their contribution to this paper.

## Author contributions

**Conceptualization:** Xiangrui Guo, Xuedong Song.

**Data curation:** Dingli Chen, Cairu Liu, Ruimin Zhang, Yongxue Chen, Xiangrui Guo, Xuedong Song.

**Formal analysis:** Shurui Li, Subin Zhao, Cairu Liu, Ruimin Zhang, Yongxue Chen, Xiangrui Guo, Xuedong Song.

**Investigation:** Shouxia Li, Shurui Li, Dingli Chen, Subin Zhao, Cairu Liu, Ruimin Zhang, Yongxue Chen, Xiangrui Guo.

**Methodology:** Dingli Chen, Subin Zhao, Cairu Liu, Xiangrui Guo.

**Resources:** Shouxia Li, Shurui Li, Dingli Chen, Subin Zhao, Cairu Liu, Ruimin Zhang, Yongxue Chen.

**Software:** Yongxue Chen, Xiangrui Guo.

**Supervision:** Shouxia Li, Shurui Li.

**Validation:** Shouxia Li.

**Visualization:** Shouxia Li.

**Writing—original draft:** Shouxia Li, Shurui Li, Xuedong Song.

**Writing—review & editing:** Xuedong Song.

## Supplementary Material


