# Supplementary material for: Case report of a novel mutation in the TNC gene in Chinese patients with nonsyndromic hearing loss
Source: Medicine (Baltimore). 2024 Apr 19;103(16):e37702. doi: 10.1097/MD.0000000000037702 (PMC11029965; doi:10.1097/MD.0000000000037702)
Supplement: Supplementary file 1 [file medi-103-e37702-s001.docx]

**Supplementary Material**

**Website and database information to be accessed**

1. The World Health Organization (WHO): Home/Newsroom/Fact sheets/Detail/Deafness and hearing loss.

<https://www.who.int/en/news-room/fact-sheets/detail/deafness-and-hearing-loss>.

2. Morl Lab at the University of Iowa: to date, 224 genes have been reported to be associated with hearing loss.

<https://morl.lab.uiowa.edu/genes-included-otoscope-v9>.

3. Hereditary Hearing Loss Homepage:

<https://hereditaryhearingloss.org/>

4. National Center for Biotechnology Information. ClinVar:

<https://www.ncbi.nlm.nih.gov/clinvar/variation/VCV000227980.38>

5. SIFT:

<http://sift.bii.a-star.edu.sg/>

6. PolyPhen-2:

<http://genetics.bwh.harvard.edu/pph2/index.shtml>

8. Mutation Taster:

<http://www.mutationtaster.org/>

9. AlphaFold's online prediction program:

<https://alphafold.ebi.ac.uk/>

10. SWISS MODEL:

<http://swissmodel.expasy.org/>
